# The role of *Fragaria vesca* homolog of a (Z)-3:(E)-2-hexenal isomerase in the development of green-leafy fruit aroma

**DOI:** 10.1093/hr/uhaf163

**Published:** 2025-06-26

**Authors:** Rong Zhang, Dylan Nunnally Martínez, Elli A Koskela, Amparo Monfort

**Affiliations:** Centre for Research in Agricultural Genomics (CRAG), CSIC-IRTA-UAB-UB, Edifici CRAG, Campus UAB, 08193 Bellaterra, Barcelona, Spain; Centre for Research in Agricultural Genomics (CRAG), CSIC-IRTA-UAB-UB, Edifici CRAG, Campus UAB, 08193 Bellaterra, Barcelona, Spain; Centre for Research in Agricultural Genomics (CRAG), CSIC-IRTA-UAB-UB, Edifici CRAG, Campus UAB, 08193 Bellaterra, Barcelona, Spain; VTT Technical Research Centre of Finland, Edifici CRAG, Campus UAB, Tekniikantie 21, 00240 Espoo, Finland; Centre for Research in Agricultural Genomics (CRAG), CSIC-IRTA-UAB-UB, Edifici CRAG, Campus UAB, 08193 Bellaterra, Barcelona, Spain; IRTA, Genomics and Biotechnology, Edifici CRAG, Campus UAB, 08193 Bellaterra, Catalonia, Spain

## Abstract

The green leaf volatiles (Z)-3-hexenal and (E)-2-hexenal are key components of the characteristic strawberry aroma, an important determinant of consumer preferences. Green leaf volatiles (GLVs) are C6 compounds that impart fresh, green notes and are involved in plant wounding responses. GLV biosynthesis requires several enzymatic steps to convert polyunsaturated fatty acids to C6-aldehydes, alcohols, and esters, respectively. However, the biosynthesis of GLVs in strawberries, such as the isomerization of (Z)-3-hexenal to (E)-2-hexenal, remains poorly understood. In this study, we identified a (Z)-3:(E)-2-hexenal isomerase gene (*FvHI*) using phylogenetic analysis, characterized its expression in different tissues, and characterized its function using stable transformation. Volatile analysis by gas chromatography–mass spectrometry (GC–MS) of fruits from a *Fragaria vesca* near-isogenic line (NIL) collection revealed a distinct ratio of (Z)-3-hexenal and (E)-2-hexenal in lines containing *Fragaria bucharica* introgressions in the distal end of linkage group 5. Consequently, *FvHI* was located within this genomic region. The coding sequence of *FvHI* was nearly identical between the recurrent parent and a selected NIL individual containing an introgression in the distal end linkage group 5, indicating that the contrasting ratio of (Z)-3 and (E)-2 GLV isomers may be attributed to transcriptional differences. Accordingly, *FvHI* expression in ripe fruits was lower in the selected NIL individual than in the recurrent parent. Lastly, *FvHI* overexpression decreased (Z)-3-hexenal accumulation and increased (E)-2-hexenal accumulation in the recurrent parent and the selected NIL individual. These results suggest that *FvHI* plays a role in producing the characteristic strawberry aroma by converting (Z)-3-hexenal to (E)-2-hexenal.

## Introduction

During the last 10 years, consumer demand for better-tasting fruit and produce has increased dramatically, and garden strawberries (*Fragaria* x *ananassa* Duch.) are not an exception. Strawberry taste is a complex trait formed by an interaction of fruit texture, sugar content, acidity, and volatile organic compounds (VOCs). Of these attributes, fruit sugars and volatile composition are the factors most closely associated with overall liking [1]. Consequently, researchers and strawberry breeders have identified a handful of candidate genes and molecular markers that contribute to the accumulation of specific VOCs to speed up breeding for better-tasting strawberries. For instance, genetic mapping experiments suggest the strawberry fatty acid desaturase gene (*FaFAD1)* is associated with the accumulation of gamma-decalactone, which is responsible for peach-like fruity aroma [2, 3]. Furthermore, a mutation in a strawberry nerolidol synthase gene (*FaNES1*) has been linked to the accumulation of linalool and nerolidol, which both confer a sweet flowery aroma [4]. A strawberry anthranilic acid methyl transferase gene (*FaAAMT*) has been linked to the biosynthesis of methyl anthranilate, which contributes a grape-like fruity aroma [5]. However, the accumulation of methyl anthranilate is not solely dependent on *FaAAMT* activity and is likely a polygenic trait regulated by several distinct loci as well as environmental interactions [6].

In garden strawberry, the polygenic regulation of VOC accumulation, complex octoploid genetics, and genotype–environment interactions pose major hurdles for deciphering the genetic basis of fruit aroma. To alleviate the problem of the complex polyploid genetics in the garden strawberry, the closely related diploid woodland strawberry, *Fragaria vesca*, has been extensively used as a model species. The genomes of the garden strawberry and *F. vesca* are highly collinear, facilitating the transfer of genetic knowledge between the two species [7]. Moreover, *F. vesca* is one of the four subgenome donors of the garden strawberry and dominates the other subgenomes by having higher gene content, greater gene expression activity, and homoeologous exchanges biased towards the *F. vesca* subgenome [7]. It is of special interest to note that metabolic pathways associated with strawberry aroma are largely regulated by the *F. vesca* subgenome [7]. Therefore, the use of *F. vesca* as a model to study the genetic basis of VOC accumulation in strawberries could prove to be a very fruitful approach. The development of genetic resources in *F. vesca* has rapidly progressed during the last ten years. Importantly, there are already resources available to study the accumulation of VOCs in diploid strawberries. A near-isogenic line (NIL) collection has been developed from a cross between a *F. vesca* accession ‘Reine des Vallées’ and the diploid *F. bucharica* [8]. The NIL collection has been extensively characterized for fruit quality attributes, particularly for VOC accumulation [9]. The study by Urrutia *et al*. (2017) [9] identified several QTLs responsible for the accumulation of 19 key volatile compounds (KVCs) that are mainly responsible for the characteristic strawberry aroma [10–12]. Four large-effect QTLs for the accumulation of the KVCs (Z)-3-hexenal, (Z)-3-hexenyl acetate, (E)-2-hexenal and (E)-2-hexenyl acetate co-localized at the distal end of the linkage group 5 [9].

These compounds, as well as other C6 aldehydes, alcohols, and esters, are often termed green leaf volatiles (GLVs) as they confer a characteristic green leaf odour [10]. This odour is sometimes associated with ‘freshness’ [13] and sometimes with ‘unripeness’ [14]. Some GLVs can also enhance sensory perception of sweetness and are positively correlated with overall liking [15]. In addition, GLVs are involved in plant defence both directly and indirectly. In response to wounding, the GLV (Z)-3-hexenal is rapidly emitted, followed by (E)-2-hexenal, hexenols, and hexenyl acetates [16, 17]. Once emitted, GLVs have been shown to repel herbivorous insects, attract predatory insects, prime the defences of neighbouring plants, and influence the growth of bacteria and fungi, among other effects [17]. Furthermore, GLVs are known to play a role in phytohormonal crosstalk by influencing jasmonic acid biosynthesis and signalling [18–20].

Biosynthesis of GLVs begins in chloroplasts [21] by the addition of molecular oxygen to α-linolenic or linoleic acid. The reaction is catalysed by 13-lipoxygenases (13-LOXs; reviewed in Viswanath et al. 2020 [22]) to yield polyunsaturated fatty acids (PUFAs). PUFAs are further isomerised by hydroperoxide lyase to produce unstable hemiacetals, which spontaneously dissociate into volatile six-carbon aldehydes (Z)-3-hexenal or hexanal, from α-linolenic and linoleic acid, respectively [23]. These C6 aldehydes are the first GLVs produced by the biosynthesis pathway and are parent compounds for all other derivative GLVs comprising of C6 aldehydes, alcohols and esters. (Z)-3-hexenal may be reduced to alcohol (Z)-3-hexenol by alcohol dehydrogenases [24], followed by further conversion to the ester (Z)-3-hexenyl acetate by a member of BADH acyltransferase gene family [25]. However, (Z)-3-hexenal may also be isomerised into (E)-2-hexenal by a (Z)-3:(E)-2-hexenal isomerase [26]. (E)-2-hexenal may be reduced to the corresponding alcohol (E)-2-hexenol and further to the ester (E)-2-hexenyl acetate by the same enzymes as mentioned for (Z)-3-hexenal derivatives.

In this study, we build on the work of Urrutia *et al*. [9] to identify the gene responsible for four major-effect QTLs associated with the accumulation of GLV compounds in diploid strawberry. We show gene expression data to demonstrate differential regulation of the causal gene in the parents of the NIL collection and provide functional evidence for the role of the causal gene as the main determinant of the accumulation of (E)-2 GLV isomers in diploid strawberry.

## Results

### Volatile compounds in ripe strawberry fruits

**Figure 1:**
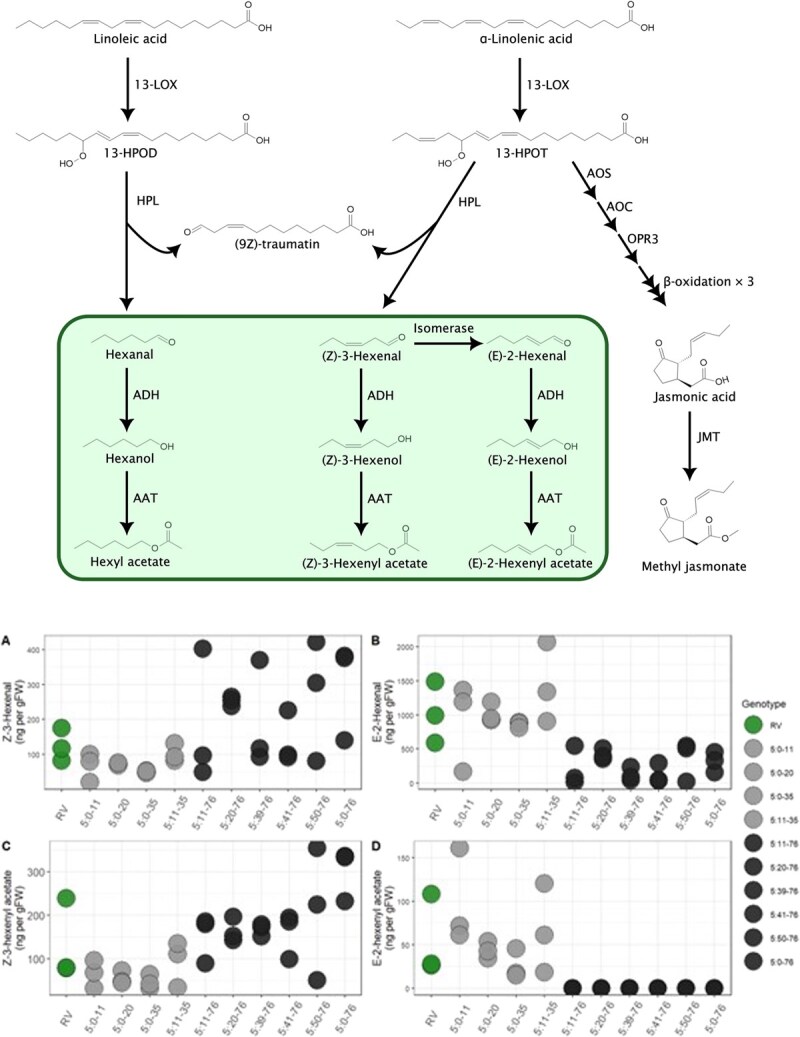
Biosynthetic pathway of Linoleic acid and accumulation of the GLV in strawberry samples A) (Z)-3-hexenal, B) (E)-2-hexenal, C) (Z)-3-hexenyl acetate, and D) (E)-2-hexenyl acetate in RV and LG5 introgression lines, determined as ng/g of fresh weight in red ripe berries. Three biological replicates for all genotypes were independently analysed on the same day they were prepared.

The NILs carrying *F. bucharica* introgressions at the distal end of LG5 have been reported to accumulate higher amounts of (Z)-3-hexenal and (Z)-3-hexenyl acetate and lower amounts of (E)-2-hexenal and (E)-2-hexenyl acetate than the recurrent parent RV [9]. We measured the accumulation of these compounds in ripe strawberry fruits by GC–MS in all NILs carrying introgressions in LG5, as well as in the recurrent parent RV (Fig. 1). Our results, in line with the results reported by Urrutia *et al.* [9], show that the NILs harbouring an introgression at the end of LG5 accumulated higher amounts of (Z)-3-hexenal and (Z)-3-hexenyl acetate than RV and also than the NILs with introgressions in the beginning or middle of LG5 (Fig. 1A and C). Likewise, the accumulation of (E)-2-hexenal and its derivative (E)-2-hexenyl acetate followed the opposite pattern, with NILs harbouring an introgression at the end of LG5 RV having lower levels of the compounds than RV and also than the NILs with introgressions in the beginning or middle of LG5 (Fig. 1B and D). Our findings confirmed the earlier reports [9] on the presence of a QTL affecting the accumulation of GLV compounds located within the *F. bucharica*-derived introgression at the end of LG5.

### *F. vesca* homologs of (Z)-3:(E)-2-hexenal isomerase

Based on our findings and earlier reports [9], the *F. bucharica* introgression within LG5:50-76 harbours a QTL that affects the accumulation of (Z)-3-hexenal and (E)-2-hexenal and their respective derivatives (Z)-3-hexenyl acetate and (E)-2-hexenyl acetate. A key step in the biosynthesis pathway of these compounds has been identified in bell pepper (*Capsicum annuum* L.), in which the isomerization of (Z)-3-hexenal into (E)-2-hexenal takes place via the activity of a (Z)-3:(E)-2-hexenal isomerase [26]. Similarly, in cucumber (*Cucumis sativus*) the conversion of (Z)-3-hexenal into (E)-2-hexenal is dependent on the activity of a cucumber (Z)-3:(E)-2-hexenal isomerase [27]. To identify *F. vesca* homologs of (Z)-3:(E)-2-hexenal isomerase (HI), we performed a BLAST query with cucumber HI amino acid sequence against the *F. vesca* protein database. This resulted in the discovery of four proteins with bit scores higher than 200 (Table S4). The protein with the highest score was FvH4_5g29270, located in the 5th chromosome within the LG5:50-76 introgression.

**Figure 2:**
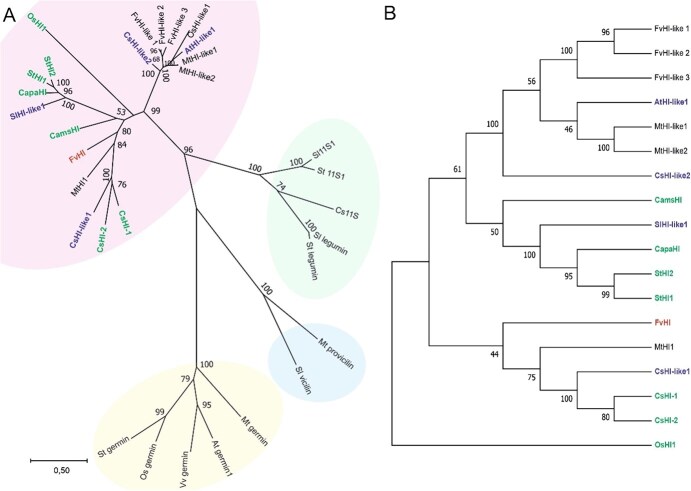
Phylogenetic relationships of putative *F. vesca* HI and HI-like proteins. A) Phylogenetic tree of cupin superfamily proteins from various plant species. Proteins belonging to HI and HI-like, 11S seed globulin, vicilin and germin clades are highlighted by red, green, blue and yellow backgrounds, respectively (a clockwise direction). B) Bootstrap consensus tree of HI- and HI-like proteins. Proteins with (CamSHI, CapaHI, StHI2, StHI1, CsHI-1, CsHI-2, OsHI1) and without (SIHI-like1, CsHI-like1, CsHI-like2) demonstrated HI activity are shown in green and blue fonts, respectively. The putative FvHI homolog is shown in red font. The values next to branching points indicate the percentage of bootstrap support with 1000 replications.

Phylogenetic relationships of the four putative *Fragaria* HI and HI-like proteins were studied by comparing the *Fragaria* proteins to other proteins of the cupin superfamily (Table S2) from various plant species. All four *Fragaria* proteins were most closely associated with the HI and HI-like protein clade (Fig. 2A). A more detailed analysis of the HI and HI-like clade revealed that only the protein FvH4_5g29270 clustered together with HI proteins possessing isomerase activity (Fig. 2B). Next, we investigated whether the identified FvHI proteins possess the amino acid residues essential for HI enzymatic activity [26]. Alignment of the predicted *F. vesca* proteins against bell pepper HI showed that only the FvH4_5g29270 protein possesses the three amino acid residues essential for hexenal isomerase activity (Fig. S2A). These data suggested that only the protein encoded by FvH4_5g29270 is an actual *Fragaria* HI, while the other three *Fragaria* proteins are similar to HI-like proteins.

As the protein FvH4_5g29270 is located within the *F. bucharica* introgression that affects the accumulation of (Z)-3-hexenal and (E)-2-hexenal, we decided to clone the gene encoding the protein from both RV and a NIL harbouring *F. bucharica* introgression to see whether the protein itself is altered or non-functional in *F. bucharica*-derived NILs. Comparing the coding sequence we identified genetic variation between *F. vesca* and the Fv5:41-76 NIL, which is derived from *F. bucharica*. The presence of seven base pair substitutions in the coding sequence show that these are two distinct alleles (Fig. S2C), one from *F. vesca* and one from donor parent *F. bucharica*. Despite the alleles differences of the gene, the predicted proteins from RV and the NIL LG5:41-76 were highly similar with only three conservative changes (Fig. S2B). These data suggested that if the hexenal isomerase gene FvH4_5g29270 (referred to as *FvHI* hereafter) is the causative agent behind the altered (Z)-3:(E)-2-hexenal conversion rate observed in near-isogenic lines harbouring an *F. bucharica* introgression, the difference probably occurs at the transcriptional level.

### Gene expression patterns of *FvHI* in RV and near-isogenic lines

**Figure 3:**
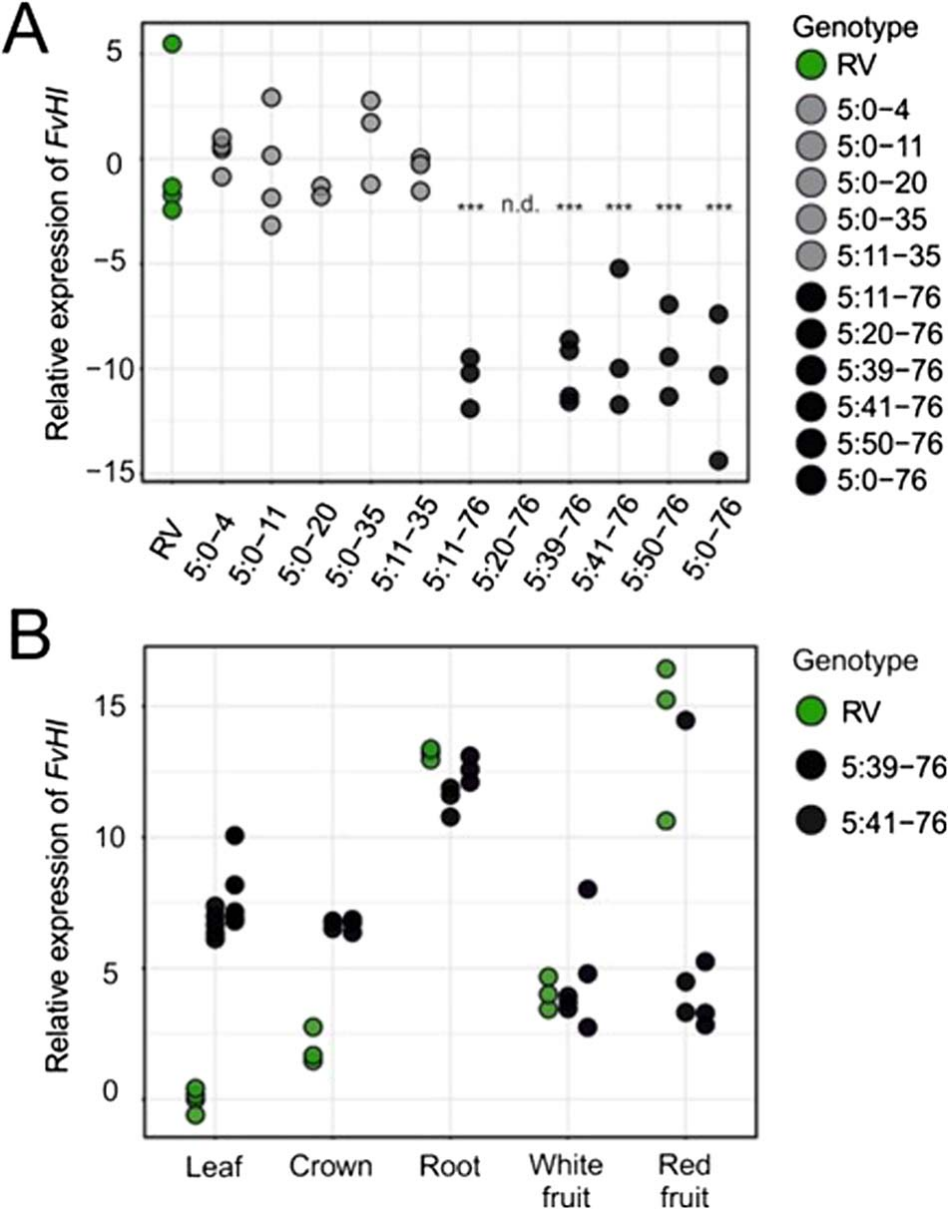
Relative expression of *FvHI* in different genotypes and plant tissues. A) Relative expression of *FvHI* in NILs with introgressions covering different regions of the LG5. Light grey and dark grey dots depict NILs with the RV and *F. bucharica* alleles of *FvHI*, respectively. B) Relative expression of *FvHI* in various plant tissues of RV and in two NILs with introgressions containing the *F. bucharica* allele of *FvHI*. Relative expression values have been normalized to *FvMSI1* and shown as log transformations of the fold change values. Asterisks indicate statistically significant differences as compared to RV values by Dunnett’s test: ***p < 0.001; n.d., not detected.

To elucidate if changes occur at the transcriptional level, we investigated gene expression patterns of *FvHI* in the recurrent parent RV and NILs with *F. bucharica* introgressions covering different regions of the LG5. We first analysed *FvHI* expression in fully ripe fruits of field-grown plants. The mRNA levels of the gene *FvHI* were similar in RV and in NILs covering the LG5 until 35-cM region. However, the NILs harbouring an introgression at the end of LG5 showed extremely low levels of *FvHI* mRNA (Fig. 3A). This corroborated our hypothesis that *FvHI* is the gene responsible for the low (Z)-3:(E)-2-hexenal conversion rate.

We also examined the tissue-specific expression patterns of *FvHI* in the recurrent parent RV and in two NILs with introgression at the end of LG5 (Fig. 3B). The relative expression rates in fully ripe red fruits were in concordance with the results obtained from field-grown plants; *FvHI* mRNA was significantly more abundant in RV than in the two NILs. However, the level of *FvHI* expression in white fruits was comparable in the three genotypes. Interestingly, the NILs with introgressions at the end of LG5 had higher levels of *FvHI* expression in leaves and crowns than in RV.

The observed differences in *FvHI* expression between the recurrent parent RV and the LG5 NIL without having detected differences in the coding sequence could suggest that the promoter regions of *F. bucharica FvHI* probably differ from those of RV. Therefore, we attempted to sequence the promoter regions of *FvHI* from RV and *F. bucharica* using primers designed to bind to different locations in the promoter regions (Table S3B). Primers were designed based on the reference genome V4 of *F. vesca* accession H4, which was also used to design the primer clone CDS of *FvHI*, and most of the primers were also predicted to have binding sites in the available *F. bucharica* scaffolds including *FvHI* region (*F. bucharica* draft genome, Uni. Helsinki) (Fig. S3A). Although we were able to sequence the genomic region of *FvHI* from both diploid species and show that the FvHI proteins from RV and *F. bucharica* are identical (Fig. S2B), we were unable to amplify any fragments from *F. bucharica* promoters, even after using different primer combinations, extended extension times or lowered annealing temperatures. These results suggested that the promoter region of *F. bucharica FvHI* is highly divergent from its *F. vesca* counterpart as observed in the *in silico* alignment of the *FvHI* regions of both genomes (Fig. S3B).

### Function of (Z)-3:(E)-2-hexenal isomerase in *F. vesca*

To provide functional evidence on the role of FvHI in the conversion of (Z)-3- to (E)-2-hexenal, we generated transgenic plants with constitutive 35S-driven overexpression of *FvHI* in the RV and NIL 5:50-76 genetic backgrounds. We were able to acquire five and two fertile and independent *FvHI* overexpressing lines in the RV and NIL 5:50-76 backgrounds, respectively. To examine the effect of *FvHI* overexpression on the hexenal conversion ratio, we raised the plant materials under greenhouse conditions and collected ripe red berries for gene expression and volatile compound analyses.

**Figure 4:**
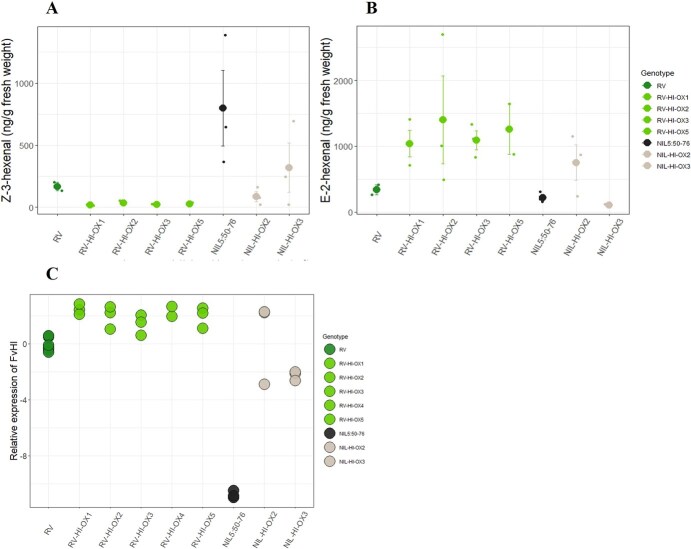
GLV accumulation and *FvHI* expression in RV, NIL 5:50-76 and their derivative transgenic lines. A) (Z)-3-hexenal and B) (E)-2-hexenal determined as ng/g of fresh weight in red ripe berries (averages are presented by larger dots, error bars present standard error (n = 3)), and C) Relative expression of *FvHI.* (FvMSI1 was used as house-keeping gene for normalizing sample-to-sample variation, Dots present expression data from independent biological replicates (n = 3)).

As in our earlier experiments, *FvHI* expression in ripe red fruits was much higher in wild-type RV than in the wild-type NIL 5:50-76. Transgenic lines carrying the *FvHI* overexpression construct in the RV background showed slightly elevated *FvHI* expression levels as compared to wild-type RV. Also, the transgenic lines in the NIL 5:50-76 background had higher *FvHI* relative expression levels than the wild-type NIL 5:50-76, but with more variation between the transgenic lines and biological replicates (Fig. 4C).

Next, we determined GLV accumulation in red ripe berries of RV, NIL 5:50-76 and their derivative transgenic lines. Overexpression of *FvHI* boosted the conversion of (Z)-3-hexenal to (E)-2-hexenal in all examined transgenic lines in the RV background, as well as in one of the two transgenic NIL 5:50-76 lines (Fig. 4). These data provide functional evidence for the role of *FvHI* as the main determinant of (Z)-3 versus (E)-2 hexenal conversion rate in *F. vesca*.

## Discussion

Here, we identify the causal gene for the GLV QTLs observed by Urrutia *et al.* [9]. We show by genetic and functional analyses that the *F. vesca* homolog of the *HI* gene has a key role in the conversion of (Z)-3-hexenal to (E)-2-hexenal. Although the *FvHI* gene exhibits allelic variation between *F. vesca* and *F. bucharica*, the high similarity between the predicted proteins suggests that the protein function is likely conserved. Therefore, if FvHI is indeed responsible for the altered (Z)-3:(E)-2-hexenal conversion ratio observed in NILs, the underlying cause is most likely due to differences in gene expression regulation, rather than changes in protein structure or function.

The gene that we have identified encodes a protein with a high level of sequence similarity to functionally validated HI proteins from a variety of plant species (Fig. 2). HI proteins with hexenal isomerase activity have been previously characterized in bell pepper [26], cucumber [27], and tea plant (*Camellia sinensis*) [28], as demonstrated by the increased proportion of (E)-2-hexenal in heterologous systems overexpressing species-specific *HI*s*.* Similarly, our results of overexpressing *FvHI* in *F. vesca* altered the GLV bouquet by increasing the proportion of (E)-2-hexenal to (Z)-3-hexenal (Fig. 4).

GLVs are also produced in large quantities in garden strawberry. Two recent studies [29, 30] showed that (E)-2-hexenal was among the most abundant VOCs measured in ripe fruits of garden strawberry. However, the QTL study by Rey-Serra *et al.* [30] was unable to identify stable QTLs for (E)-2-hexenal in the two studied mapping populations. Given that (E)-2-hexenal accumulation was overall very high in the two studied biparental mapping populations [30], it is possible that all the *F.* x *ananassa HI* variants present in the populations analysis were able to actively convert (Z)-3-hexenal to (E)-2-hexenal, resulting in minor phenotypic differences and low mapping power. In contrast, Fan *et al.* [29] used GWAS to identify four loci associated with (E)-2-hexenal accumulation, indicating a polygenic inheritance pattern. The four loci resided in homoeologous groups 3D, 4A, 6D and 7B [29] and are therefore unlikely to contain a homolog of *FvHI*, which resides in *F. vesca* LG5. The biosynthetic pathway leading to (E)-2-hexenal involves multiple enzymes, many of which belong to large protein families with multiple isoforms. For instance, lipoxygenases catalysing the first step of GLV biosynthesis are represented in the *F. vesca* genome by 14 genes [31]. It is feasible that the four loci identified by Fan *et al.* [29] are associated with GLV biosynthesis genes or their regulators. Our findings on the role of *FvHI* in (Z)-3:(E)-2 hexenal conversion could provide a new target gene for altering the GLV bouquet [15].

The interest in studying the accumulation of strawberry GLVs has been sparked by their contribution to ‘fresh’ or ‘green’ notes in strawberry fruit [11]. Formerly, these notes have often been associated with undesirable aroma characteristics such as unripeness [14]. More recent studies have associated GLVs with the ‘fresh’ note in ripe strawberry fruit [13], and (E)-2-hexenal has been shown to have a positive association with overall liking and sensory perception of sweetness [15]. It is therefore difficult to estimate how a change in the accumulation of specific compounds affects the overall aroma or sensory perception.

GLVs are important for the horticultural industry not only because of their effect on fruit aroma, but also because of their biological functions in plant defence. Enhancing GLV production by overexpressing GLV biosynthesis pathway genes leads to increased resistance against fungal pathogens (e.g., *Botrytis cinerea*) and reduces herbivore damage by attracting parasitoids [32]. Different GLVs have specific biological effects; for instance, (E)-2-hexenal has higher bactericidal activity at low concentrations than (Z)-3-hexenol both *in vivo* and *in vitro* [33]. Moreover, (E)-2-hexenal is more effective than (Z)-3-hexenal in inducing pathogen resistance by increasing cell wall lignification and activating the production of other antifungal substances in *Arabidopsis* [34]. Finally, GLVs have a role in plant-to-plant signalling. GLV production is rapidly induced in mechanically wounded *Chrysanthemum cinerariaefolium* seedlings. Within hours of being subjected to emitted GLVs, non-wounded plants activate the production of the natural insecticide pyrethrin by upregulating pyrethrin biosynthesis genes [35].

Studies on the biological effects of GLVs in strawberries are still relatively scarce. Myung *et al.* [36] demonstrated that wounding induces the biosynthesis of (Z)-3- and (E)-2-hexenal in garden strawberry. The reports on the effect of GLVs on pathogen resistance in strawberries are contradictory; while Abanda-Nkpwatt *et al*. [37] showed that C6 aldehydes can inhibit the growth of *B. cinerea in vitro,* Xu *et al.* (2021) [38] demonstrated that (E)-2-hexenal actually increases the growth of *B. cinerea* on stored strawberry fruit. The transgenic lines we report here that overexpress the *FvHI* gene could be used to clarify the effects of (Z)-3-hexenal and (E)-2-hexenal on both pathogen and herbivore resistance in strawberries.

## Materials and methods

### Plant materials

The construction of near-isogenic lines is described in detail in Urrutia *et al*. [8]. The NILs used in the current experiments are described in supplementary Table S1 and Fig. S1. In summary, 11 NILs covering different regions of the diploid *Fragaria* linkage group 5 (LG5) and their recurrent parent ‘Reine des Vallées’ (RV) were used in all experiments. All plant materials used in the experiments were maintained and propagated from seed as fixed, fully homozygous lines.

Seeds in all experiments were stored at +4°C for a minimum of 2 weeks. Seed material was subjected to 3% (w/v) Captan (ADAMA Ltd.) solution for 5 minutes followed by rinsing with autoclaved Milli-Q Ultrapure water (Merck Millipore). The seeds were further sterilized in 1 M H_2_SO_4_ for five minutes and rinsed three times with autoclaved water. The seeds were left to soak in fresh autoclaved water at room temperature overnight. The next day, the seeds were placed on filter paper moistened with autoclaved Milli-Q Ultrapure water (Merck Millipore) in Petri dishes. Petri dishes were sealed with Parafilm and placed in a growth chamber at 24°C to 28°C. After germination, seedlings were planted in Jiffy pots (Jiffy Group International, Netherlands).

Plants destined for outdoor cultivation were sown in October 2017 and grown in a greenhouse compartment at 22/17°C (day/night) without artificial lighting until March 2018. In March, the plants were transferred to a shaded greenhouse at Centre Torre Marimon in Caldes de Montbui (latitude 41°36′N, longitude 2°10′E at 203 m of altitude from sea level). Each genotype was represented by two to four individual plants. The plants did not receive additional lighting or heating. Usual agronomical practices for strawberry production were followed.

Seedlings destined for controlled climate experiments were germinated at the end of July 2018. Germinated seedlings were transferred onto soil in mid-August 2018 and cultivated in a growth chamber with a 16-hour photoperiod at 22°C for 11 weeks. After this, the plants were moved to a greenhouse compartment under a 16-hour photoperiod at 22°C.

Transgenic lines were grown *in vitro* under a 24-hour photoperiod at 20°C to 22°C. Plantlets with two to three true leaves were transplanted into 10 × 10 cm pots containing AirBoost medium (Kekkilä, Finland) and grown under greenhouse conditions with a 16-hour photoperiod at 20°C to 22°C.

### Sampling

For volatile analysis of outdoor plants, samples were harvested during multiple periods between May 2018 and July 2018. Ripe red berries from individual plants were pooled together and analysed as independent biological replicates. Pooled samples were frozen on dry ice and stored at −75°C until volatile analysis.

Samples for RNA extraction from outdoor-grown plants were collected when the fruits were at the ripe red stage. Each genotype was represented by at least six biological replicates. The samples were frozen on dry ice and stored at −75° until RNA extraction.

RNA samples for studying tissue-specific expression patterns were collected from plants grown in a growth chamber under long-day (LD) conditions for 11 weeks. Each genotype was represented by three individual plants. For leaf tissue, the youngest fully opened leaf was sampled. For root samples, 2-cm root tips from actively growing roots were pooled together. Each plant had multiple branch crowns, and for crown samples, three branch crowns from each plant were pooled together. Fruits were sampled in the white stage and the fully ripe red stage. All samples were immediately frozen in liquid nitrogen and stored at −75°C until RNA extraction.

Fruit samples from transgenic lines were collected when the fruits were at the ripe red stage and stored at −80°C. The fruits were ground in vials precooled with liquid nitrogen in a Retsch ball mill until fine powder. Fruit powder was stored at −80°C until further processing by RNA extraction or volatile analysis.

### Analysis of volatile compounds

To prepare samples for GC–MS analysis, 1 g of frozen powdered fruit was added into a 10 ml screw-top GC–MS headspace vial, followed by the addition of 1 ml of saturated NaCl solution containing 10 ppm of internal standard (3-hexanone). The vial was immediately closed with a metal screw cap and silicone/polytetrafluoroethylene (PTFE) septum. Three biological replicates were independently analysed on the same day they were prepared.

A G6501B GC Sampler 80 (Agilent Technologies, CA, United States) was used for incubation, desorption, and extraction of volatiles. Vials were agitated at 500 rpm and incubated at 30°C for 10 minutes. Following this, an SPME fibre (50/30 μm DVB/CAR/PDMS; Supelco, PA, United States) was exposed to the headspace of the vial for 30 minutes at 30°C and 500 rpm to extract the volatiles of interest. Extracted volatiles were desorbed at the GC injection port in splitless mode at 250°C for 5 minutes. Subsequently, volatile compounds were analysed by GC–MS using a 7890A gas chromatograph coupled to a 5975C mass spectrometer with a triple-axis detector (Agilent Technologies, CA, United States). For chromatographic separation of volatiles, a DB-5MS UI GC column (60 m, 0.25 mm, 1 μm; Agilent Technologies, CA, United States) and a constant helium flow of 1.2 ml/min was used. The oven temperature started at 40°C for 2 min, increasing by 5°C/min ramp until reaching 250°C, which was maintained for 5 minutes. For the mass spectrometer, an electron ionization source was used with an ionization energy of 70 eV.

Raw GC–MS data of GLV compounds were manually integrated using Enhanced ChemStation software and compared with the NIST08 and NIST11 mass spectra libraries to predict the identity of compounds (Agilent Technologies, CA, United States). For further validation, the retention time and mass spectra of each GLV compound were compared with that of commercial standards of (E)-2-hexenal, (Z)-3-hexenal, hexanal, (E)-2-hexen-1-ol Z-3-hexen-1-ol, 1-hexanol, (E)-2-hexenyl acetate, (Z)-3-hexenyl acetate, and hexyl acetate (Sigma-Aldrich, MO, USA). For quantification of GLVs, integrated peak areas were normalized by comparison with the peak area of the internal standard (3-hexanone).

### Identification of F. Vesca (Z)-3:(E)-2-hexenal isomerase genes

The amino acid sequence of cucumber (Z)-3:(E)-2-hexenal isomerase (HI) (accession number XP_004151504.1) was used to query the *F. vesca* genome V4 protein database [39] by BLASTp with default settings (blastp -max_target_seqs -evalue 0.001 -word_size 3 -gapopen 7 -gapextend 2 -culling_limit 0 -matrix PAM30). Matches with bit scores greater than 200 were reported.

For studying the phylogenetic relationships of hexenal isomerase-like proteins, the amino acid sequences of cupin superfamily proteins from various plant species (accession numbers in Table S2) were aligned by MAFFT [40] using the iterative refinement method E-INS-I with default settings and retaining gappy regions. The HI and HI-like amino acid sequences were aligned using MAFFT [40] using the G-INS-I method with default settings and retaining gappy regions. The resulting amino acid alignments were used as input for MEGA11 [41] software. A phylogenetic tree was constructed using a maximum likelihood algorithm with default settings and 1000 bootstrap replications.

*F. vesca* and *C. annuum* amino acid sequences were aligned by MAFFT with default settings. The resulting amino acid alignments were used as input for the Color Align Properties tool of the DNA Sequence Manipulation Suite [42].

The coding sequence of *FvHI* (gene FvH4_5g29270) was amplified from leaf-derived cDNA of RV and NIL LG5:41–76 using the cloning primers described in Table S3. The resulting fragments were cloned into the TOPO-TA vector (ThermoFisher Scientific) following the manufacturer’s instructions and the inserts were sequenced using vector-specific primers.

### RNA extraction and qRT-PCR

For RNA extraction from root, leaf and crown tissues, the samples were ground into a fine powder in 2 ml Eppendorf tubes using 3 mm steel balls in a Retsch MM400 ball mill (Retsch GmbH, Germany). Samples were ground for 30 seconds at 30 Hz. Fruit samples (both white and ripe red stage) were ground for 2 minutes. The sample blocks were precooled in liquid nitrogen and care was taken not to thaw the samples while grinding. RNA was extracted using a modification of the CTAB method originally described by Monte and Somerville, (2002). Briefly, 800 μl prewarmed (65°C) CTAB buffer consisting of 2% (w/v) CTAB, 2% (w/v) PVP40, 1 M NaCl, 100 mM Tris (pH 8.0), 1 mM EDTA (pH 8.0) and 0,5 g/l spermidine supplemented with 2% (v/v) β-mercaptoethanol was mixed with the homogenized sample, extracted twice with chloroform:IAA (24:1) and precipitated overnight at +4°C in the presence of 1/3 volume of 8 M LiCl. The next day, samples were pelleted by centrifugation, dissolved in 500 μl SSTE buffer (1.0 M NaCl, 10 mM Tris–HCl [pH 8], 1 mM EDTA [pH 8] and 0.5% SDS), extracted once with chloroform:IAA and precipitated overnight at −20°C with absolute ethanol. Contaminating DNA was removed by DNAse I treatment following the manufacturer’s instructions (ThermoFisher Scientific). DNAse I was removed by chloroform:IAA extraction, followed by overnight RNA precipitation at −20°C with 1/10 volume of 3 M NaCl and absolute ethanol. Prior to cDNA synthesis, all samples were diluted to an approximate concentration of 100 ng/μl.

cDNA was synthesized from 500 ng of total RNA using PrimeScript reverse transcriptase according to the manufacturer’s instructions (TaKaRa Bio Europe S.A.S). The synthesized cDNA was diluted 5-fold prior to quantitative real-time PCR (RT-qPCR). RT-qPCR was carried out on a LightCycler 480 instrument (Roche, Mannheim, Germany) in a 10 μl total volume with a final concentration of 1x SYBR Green I Master Mix (Roche), 0.45 μm primers and 3.5 μl of diluted cDNA. All samples were run with three technical replicates. The RT-qPCR temperature profile has been described in Koskela *et al.* (2016) [43]. The primers used for RT-qPCR are described in Table S3. Relative expression levels were calculated using the 2^-ΔΔCt^ method [44] using *FvMSI1* as a normalization gene. Log-transformed relative expression values were used for statistical tests.

### *Agrobacterium*-mediated stable transformation

The coding sequence of the RV *FvHI* (gene FvH4_5g29270) was amplified in the TOPO-TA vector using Gateway-compatible gene-specific primers (Table S3) and adapter primers following the two-step *attB* adapter protocol (ThermoFisher Scientific). The amplified coding sequence was inserted into the pK7WG2D overexpression vector [45] by sequential BP and LR reactions (ThermoFisher Scientific). The identity and integrity of the insert were confirmed by sequencing.

The *FvHI* CDS in the pK7WG2D overexpression vector was transformed into the *Agrobacterium* strain GV3101. *Agrobacterium* cultures were used for the transformation of RV and LG5:50-76 NIL leaf discs as described by Oosumi et al. (2006) [46]. The transformation of regenerated plantlets was confirmed by observing GFP fluorescence under a stereo microscope.

## Supplementary Material

Web_Material_uhaf163

## Data Availability

All data is available in the tables and figures within the manuscript and supplementary material.
