# Supplementary material for: The role of Fragaria vesca homolog of a (Z)-3:(E)-2-hexenal isomerase in the development of green-leafy fruit aroma
Source: Hortic Res. 2025 Jun 26;12(10):uhaf163. doi: 10.1093/hr/uhaf163 (PMC12528648; doi:10.1093/hr/uhaf163)
Supplement: Web_Material_uhaf163 [file web_material_uhaf163.zip › Figure S3 rev.pdf]

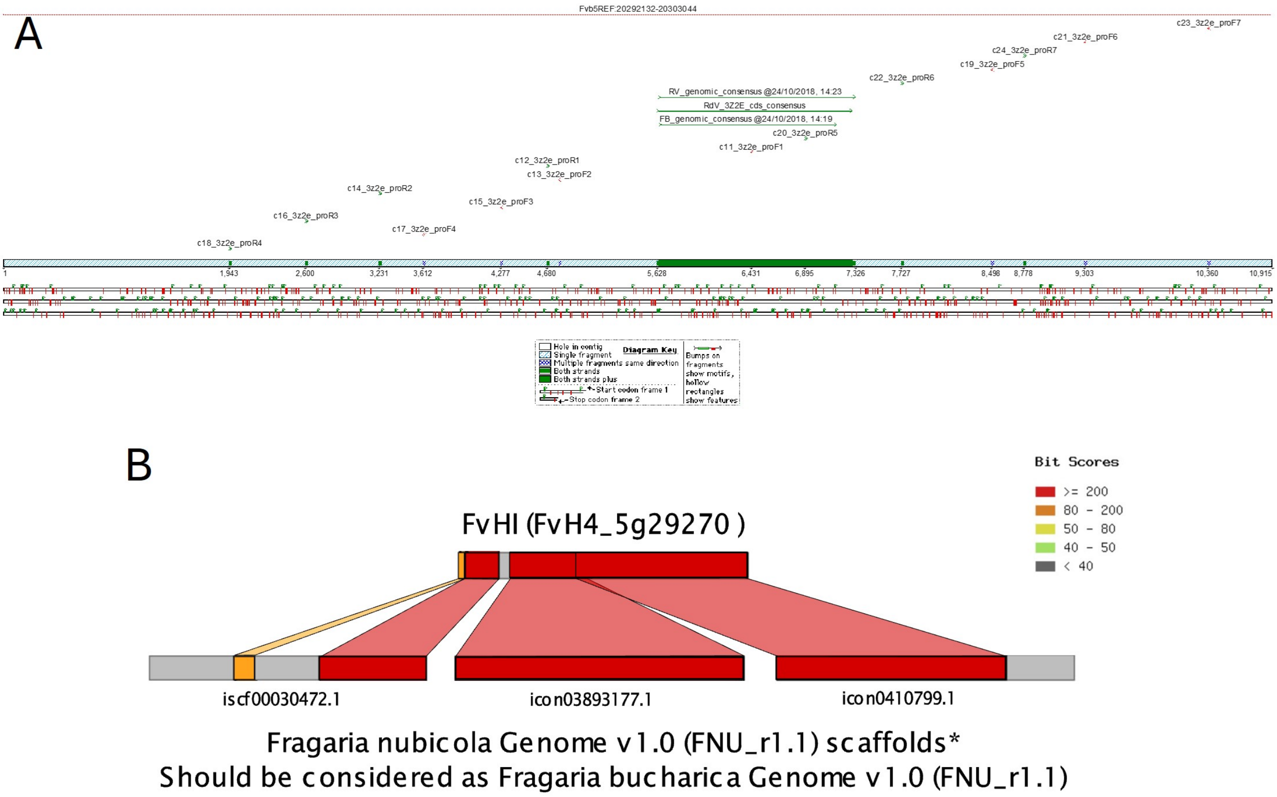

A
Fvb5REF:20292132-20303044
c23_3z2e_proF7
c21_3z2e_proF6
c24_3z2e_proR7
c19_3z2e_proF5
c22_3z2e_proR6
RV_genomic_consensus @24/10/2018, 14:23
RdV_3Z2E_cds_consensus
FB_genomic_consensus @24/10/2018, 14:19
c20_3z2e_proR5
c11_3z2e_proF1
c12_3z2e_proR1
c13_3z2e_proF2
c14_3z2e_proR2
c15_3z2e_proF3
c16_3z2e_proR3
c17_3z2e_proF4
c18_3z2e_proR4
1
1,943
2,600
3,231
3,612
4,277
4,680
5,628
6,431
6,895
7,326
7,727
8,498
8,778
9,303
10,360
10,915
Diagram Key
Hole in contig
Single fragment
Multiple fragments same direction
Both strands
Both strands plus
Start codon frame 1
Stop codon frame 2
Bumps on fragments show motifs, hollow rectangles show features
B
FvHI (FvH4_5g29270 )
Bit Scores
>= 200
80 - 200
50 - 80
40 - 50
< 40
iscf00030472.1
icon03893177.1
icon0410799.1
Fragaria nubicola Genome v1.0 (FNU_r1.1) scaffolds*
Should be considered as Fragaria bucharica Genome v1.0 (FNU_r1.1)
