# Supplementary material for: The role of Fragaria vesca homolog of a (Z)-3:(E)-2-hexenal isomerase in the development of green-leafy fruit aroma
Source: Hortic Res. 2025 Jun 26;12(10):uhaf163. doi: 10.1093/hr/uhaf163 (PMC12528648; doi:10.1093/hr/uhaf163)
Supplement: Web_Material_uhaf163 [file web_material_uhaf163.zip › Figure_S2 rev.pdf]

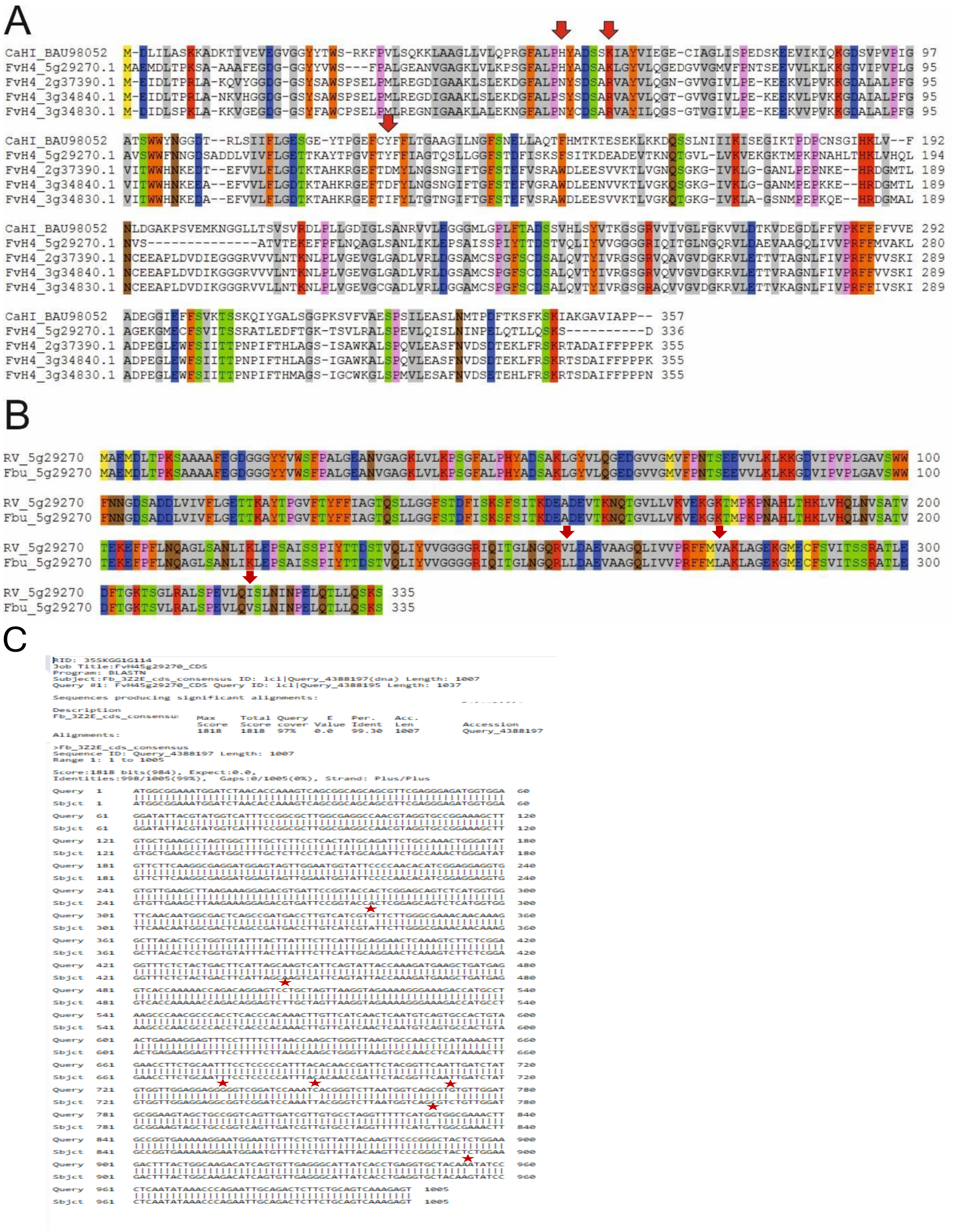
A
CaHI_BAU98052
FvH4_5g29270.1
FvH4_2g37390.1
FvH4_3g34840.1
FvH4_3g34830.1
B
RV_5g29270
Fbu_5g29270
C
