# Supplementary material for: The role of Fragaria vesca homolog of a (Z)-3:(E)-2-hexenal isomerase in the development of green-leafy fruit aroma
Source: Hortic Res. 2025 Jun 26;12(10):uhaf163. doi: 10.1093/hr/uhaf163 (PMC12528648; doi:10.1093/hr/uhaf163)
Supplement: Web_Material_uhaf163 [file web_material_uhaf163.zip › Supplement_rev2.docx]

# The role of *Fragaria vesca* homolog of a (Z)-3:(E)-2-hexenal isomerase in the development of green-leafy fruit aroma

Supplementary data

Supplementary Figures

Figure S1. Graphical representation of the LG5 NILs used in the experiments.

Figure S2. Amino acid and cDNA alignments of HI proteins from RV and *F. bucharica*.

Figure S3. Schema promotor amplification.

Supplementary Tables

Table S1. Near-isogenic lines used in the experiments.

Table S2. Accession numbers of proteins used in phylogenetic tree construction.

Table S3A. Primers used in the experiments.

Table S3B. Primers used to promotor amplification.

Table S4. *F. vesca* proteins with the highest similarity to cucumber HI.


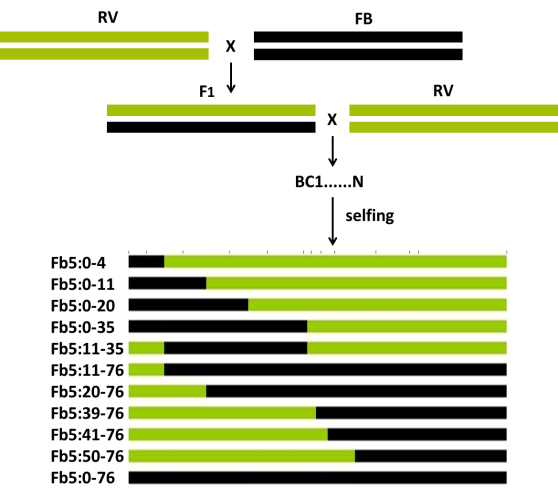


Figure S1. Graphical representation of the LG5 NILs used in the experiments.


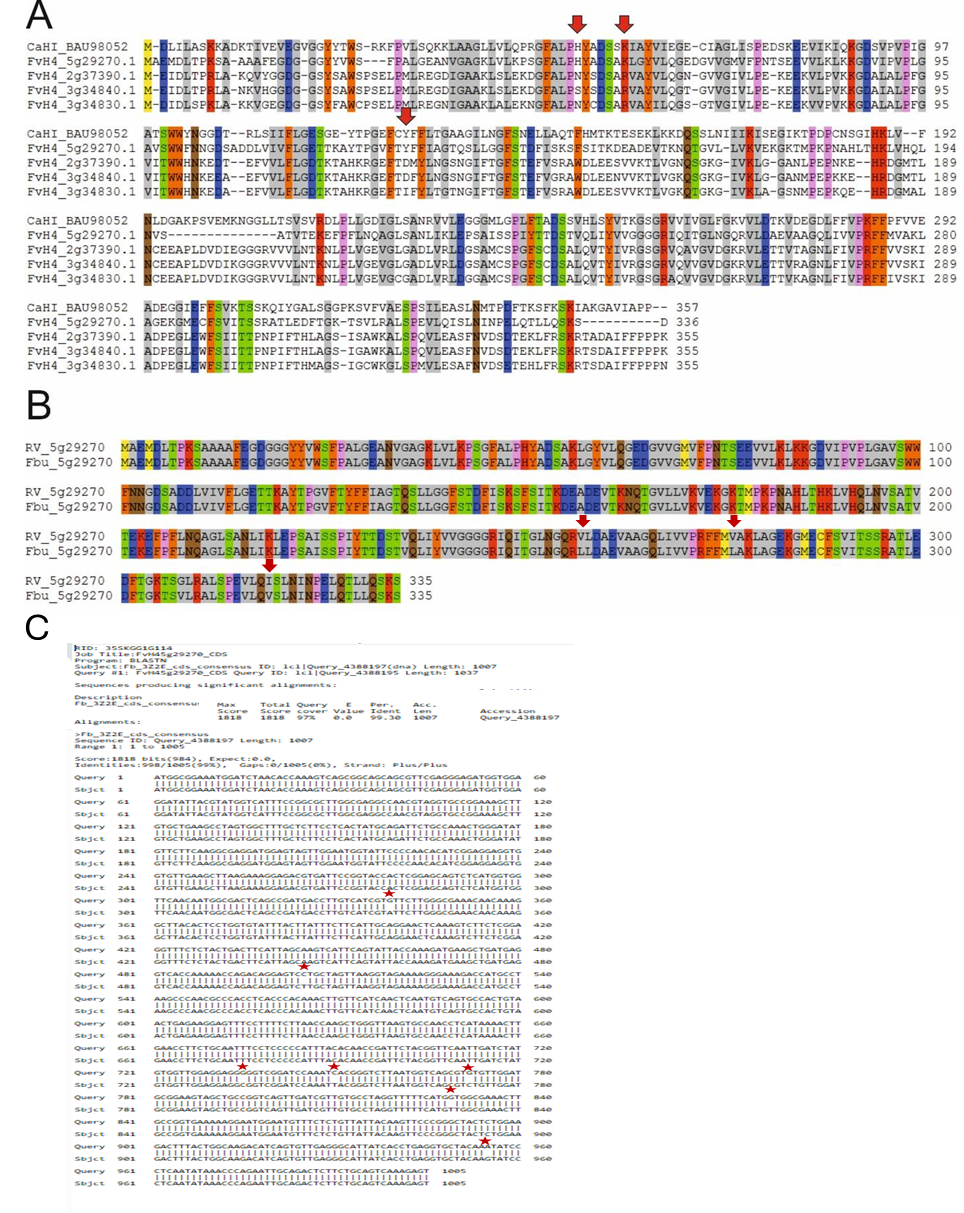


Figure S2. Amino acid alignments of HI and HI-like proteins from *F. vesca* and *F. bucharica*. A) Alignment of four *F. vesca* HI proteins with bell pepper HI (CaHI_BAU98052). The three functionally essential amino acids (H - K- Y) are highlighted by red arrows. Only the *Fragaria* protein 5g29270 possesses all three essential amino acids. B) Alignment of translated coding sequences from the recurrent parent RV and the donor parent *F. bucharica*. Amino acid differences are highlighted by red arrows. C) Alignment of coding sequences (differences highlighted by stars).

**
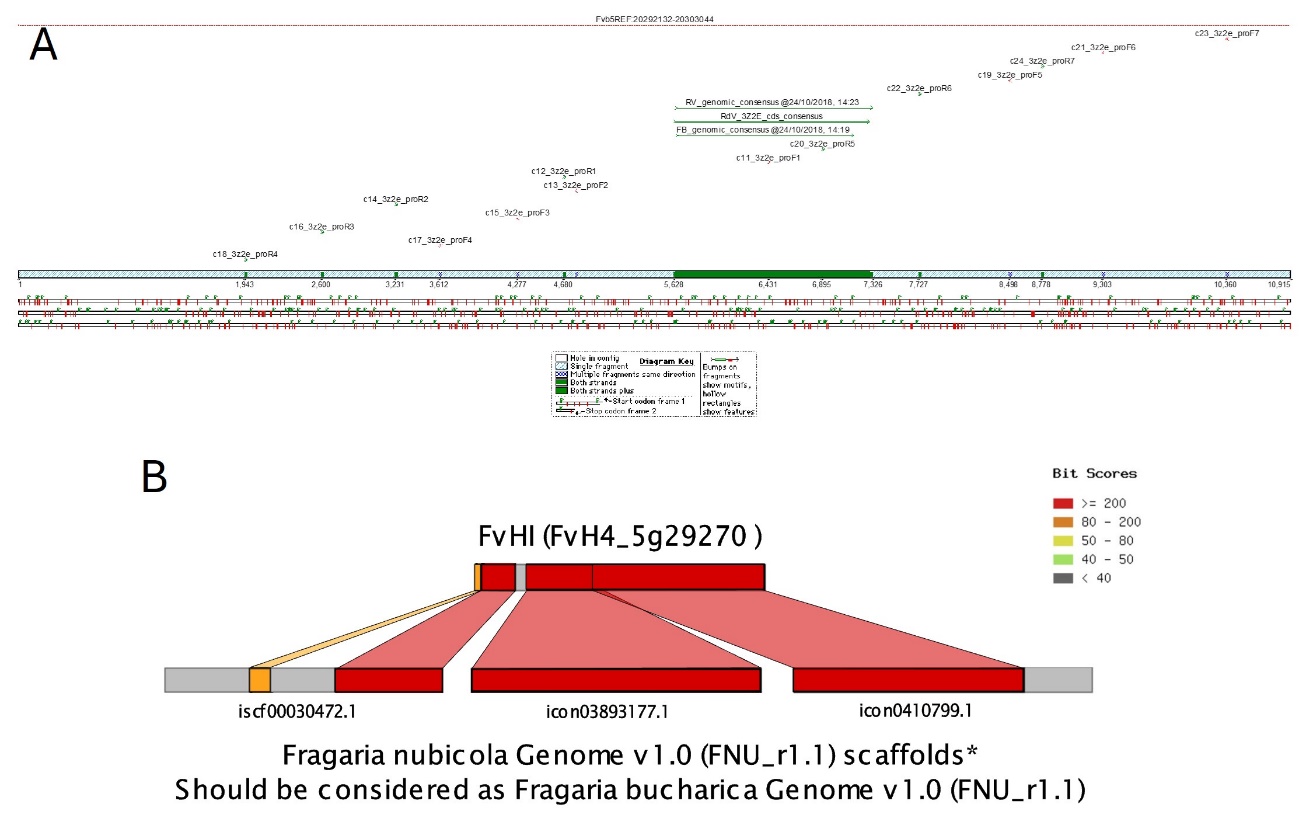
**

Figure S3. A) Schema promotor amplification. B) Alignment between FvHI regions on *F. vesca* and *F. bucharica* genomes. Reconstruction of BLAST result using the genetic sequence of the *F. vesca* hexenal isomerase gene (FvH4_5g29270) against the *F. nubicola* Genome v1.0 (FNU_r1.1) scaffolds* from the Genome Database for Rosaceae (GDR), in order to improve visualization.

*The genome was reported as *F. nubicola* in Hirakawa et al 2014 based on a USDA accession CFRA 522= PI551853. This accession was originally distributed as *F. nubicola* but subsequently re-identified as *F. bucharica* (Tennessen et al., 2014, <doi:10.1093/gbe/evu261>; <https://www.rosaceae.org/Analysis/500>)

Table S1. Near-isogenic lines used in the experiments.

| NIL name | 1^st^ marker (reference) | 1^st^ marker position on LG5 (Mb*) | 2^nd^ marker (reference) | 2^nd^ marker position on LG5 (Mb*) |
| --- | --- | --- | --- | --- |
| LG5:0-4** | CFV-3072 | 0.06 | EMFvi108 | 0.05 |
| LG5:0-11 | CFV-3072 | 0.06 | CFV-3132 | 1.4 |
| LG5:0-20 | CFV-3072 | 0.06 | CEL2 | 2.6 |
| LG5:0-35 | CFV-3072 | 0.06 | FvH4095 | 5.7 |
| LG5:11-35 | CFV-3132 | 1.4 | FvH4095 | 5.7 |
| LG5:11-76 | CFV-3132 | 1.4 | EMFv024 | 23.0 |
| LG5:20-76 | CEL2 | 2.6 | EMFv024 | 23.0 |
| LG5:39-76 | ? |  | EMFv024 | 23.0 |
| LG5:41-76 | UDF009 | 8.6 | EMFv024 | 23.0 |
| LG5:50-76 | ? |  | EMFv024 | 23.0 |
| LG5:0-76 | CFV-3072 | 0.06 | EMFv024 | 23.0 |

*megabase pairs of the *F. vesca* genome V4, pseudochromosome 5. Marker locations were determined by using the primer sequences as queries for a BLAST search against the *F. vesca* genome V4

** NIL nomenclature is based on the linkage group positions (in cMs) of the flanking markers.

Table S2. Accession numbers for proteins used for phylogenetic tree construction.

| Abbreviation | Plant species | Accession number | HI activity demonstrated | Reference |
| --- | --- | --- | --- | --- |
| AtGermin1 | *Arabidopsis thaliana* | NP_187070.1 |  |  |
| AtHI-like1 | *Arabidopsis thaliana* | NP_180436.1 | inactive | Kunishima et al. (2016) |
| CapaHI | *Capsicum annuum* | XP_016539087.1 | active | Kunishima et al. (2016) |
| CamsHI | *Camellia sinensis* | XP_028056226 | active | Chen et al. (2022) |
| Cs11S | *Cucumis sativus* | XP_011651441.2 |  |  |
| CsHI-1 | *Cucumis sativus* | XP_004151504.1 | active | Spyropoulou et al. (2017) |
| CsHI-2 | *Cucumis sativus* | Cucsa.078390 * | active | Spyropoulou et al. (2017) |
| CsHI-like 1 | *Cucumis sativus* | Cucsa.033080 * | inactive | Spyropoulou et al. (2017) |
| CsHI-like 2 | *Cucumis sativus* | XP_004150394.1 | inactive | Spyropoulou et al. (2017) |
| FvHI | *Fragaria vesca* | FvH4_5g29270** | active | This work |
| FvHI-like1 | *Fragaria vesca* | FvH4_2g37390** |  |  |
| FvHI-like2 | *Fragaria vesca* | FvH4_3g34840** |  |  |
| FvHI-like3 | *Fragaria vesca* | FvH4_3g34830** |  |  |
| MtGermin | *Medicago truncatula* | XP_013470283.1 |  |  |
| MtHI-like1 | *Medicago truncatula* | XP_003607149.1 |  |  |
| MtHI-like2 | *Medicago truncatula* | XP_003605501.1 |  |  |
| MtHI1 | *Medicago truncatula* | XP_003629975.1 |  |  |
| MtVicilin | *Medicago truncatula* | XP_003624146.3 |  |  |
| OsGermin | *Oryza sativa* | NP_001395987.1 |  |  |
| OsHI1 | *Oryza sativa* | NP_001063876.1 | active | Kunishima et al. (2016) |
| OsHI-like1 | *Oryza sativa* | XP_015639453.1 |  |  |
| SlHI-like1 | *Solanum lycopersicum* | XP_004229944.1 | inactive | Kunishima et al. (2016) |
| Sl11S1 | *Solanum lycopersicum* | XP_004247523.1 |  |  |
| SlLegumin | *Solanum lycopersicum* | XP_004234041.1 |  |  |
| SlVicilin | *Solanum lycopersicum* | NP_001308118 |  |  |
| St11S1 | *Solanum tuberosum* | XP_006351693.1 |  |  |
| StGermin | *Solanum tuberosum* | NP_001275369.1 |  |  |
| StHI1 | *Solanum tuberosum* | XP_006349431.1 | active | Kunishima et al. (2016) |
| StHI2 | *Solanum tuberosum* | XP_006349432.1 | active | Kunishima et al. (2016) |
| StLegumin | *Solanum tuberosum* | XP_006356113.1 |  |  |
| VvGermin | *Vitis vinifera* | NP_001267944.1 |  |  |

Accession numbers in the NCBI database unless stated otherwise;

* accession number from Phytozome 13 Plant Genomics Database;

** accession number from Genome Database for Rosaceae, *Fragaria vesca* genome V4.a2

Table S3A. Primers used in the experiments.

| Primers for | Forward primer | F sequence | Reverse primer | Reverse sequence | Published in |
| --- | --- | --- | --- | --- | --- |
| Amplifying *FvHI* for TOPO cloning | 5G29270-F | ATGGCGGAAATGGATCTAACACC | 5G29270-R | ACAGACTTCGATTGATGGTGCAGG | This work |
| qRT-PCR | MSI1-F | TCTCCACACCTTTGATTGCCA | MSI1-R | ACACCATCAGTCTCCTGCCAAG | Mouhu et al (2009) |
| qRT-PCR | Z3E2-F | GAGGGAGATGGTGGAGGATA | Z3E2-R | ACCACCTCCTCCGATGTGT | This work |
| Gateway primers for overexpression | attB1_5G29270-F | AAAAAGCAGGCTTCGAAGGAGATAGAACCATGGCGGAAATGGATCTAACACC | attB2_5G29270-R | AGAAAGCTGGGTACAGACTTCGATTGATGGTGCAGG | This work |

Table S3B. Primers used to promotor amplification, designed in this work.

| Forward primer | F sequence | Reverse primer | Reverse sequence |
| --- | --- | --- | --- |
| c11_3z2e_proF1 | TTGAACCACCATGAGACTGC | c12_3z2e_proR1 | CATCATCAGGCAACAACAGG |
| c13_3z2e_proF2 | TTCTAGACGCCAACGATGC | c14_3z2e_proR2 | TGGGGTATCGTTTCCTACTCC |
| c15_3z2e_proF3 | GAAAATCGGCCTCTTACTCG | c16_3z2e_proR3 | ATACACCTGCCCTCTGTTCC |
|  |  | c18_3z2e_proR4 | GGAACCACTCGTTCACAAGC |
| c19_3z2e_proF5 | GACTGCGAGAGTTCGAGAGC | c20_3z2e_proR5 | CACGGGTCTTAATGGTCAGC |
| c21_3z2e_proF6 | CTGAAAGCTCGACACACTGC | c22_3z2e_proR6 | AAGTATTGGCCAGGATTTGG |
| c23_3z2e_proF7 | GTCCGATGACAACTCAATGC | c24_3z2e_proR7 | AAGGCTCTTCCTGGAATTGG |
| c27_3z2e_proF8 | ACTCGGCGATGAGTGTGAG | c25_3z2e_proR8 | GGTTGAACGGGTGAGACC |
|  |  | c26_3z2e_proR9 | CTCGTATACACCTGCCCTCTG |
|  |  | c28_3z2e_proR10 | CTTCTTGGAATTGGGGTTTC |

Table S4. *F. vesca* proteins with the highest similarity to cucumber (Z)-3:(E)-2-hexenal isomerase.

| BLAST hit | e-value | Location in *F. vesca* genome V4 |
| --- | --- | --- |
| FvH4_5g29270 | 2.123 e-97 | chr5: 20294931..20297997 |
| FvH4_2g37390 | 2.098 e-67 | chr2: 27271351..27272846 |
| FvH4_3g34840 | 3.436 3-67 | chr3: 30118297..30121482 |
| FvH4_3g34830 | 2.290 e-63 | chr3: 30110113..30114706 |
