# Supplementary material for: The role of Fragaria vesca homolog of a (Z)-3:(E)-2-hexenal isomerase in the development of green-leafy fruit aroma
Source: Hortic Res. 2025 Jun 26;12(10):uhaf163. doi: 10.1093/hr/uhaf163 (PMC12528648; doi:10.1093/hr/uhaf163)
Supplement: Web_Material_uhaf163 [file web_material_uhaf163.zip › Supplementari File_rev2.pdf]

The role of *Fragaria vesca* homolog of a (Z)-3:(E)-2-hexenal isomerase in the development of green-leafy fruit aroma

Supplementary data

Supplementary Figures

Figure S1. Graphical representation of the LG5 NILs used in the experiments.

Figure S2. Amino acid and cDNA alignments of HI proteins from RV and *F. bucharica*.

Figure S3. Schema promotor amplification.

Supplementary Tables

Table S1. Near-isogenic lines used in the experiments.

Table S2. Accession numbers of proteins used in phylogenetic tree construction.

Table S3A. Primers used in the experiments.

Table S3B. Primers used to promotor amplification.

Table S4. *F. vesca* proteins with the highest similarity to cucumber HI.

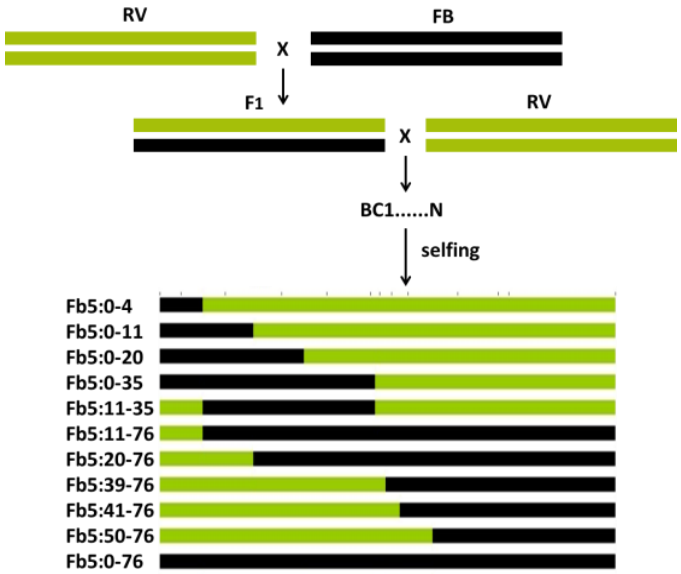

Figure S1. Graphical representation of the LG5 NILs used in the experiments.

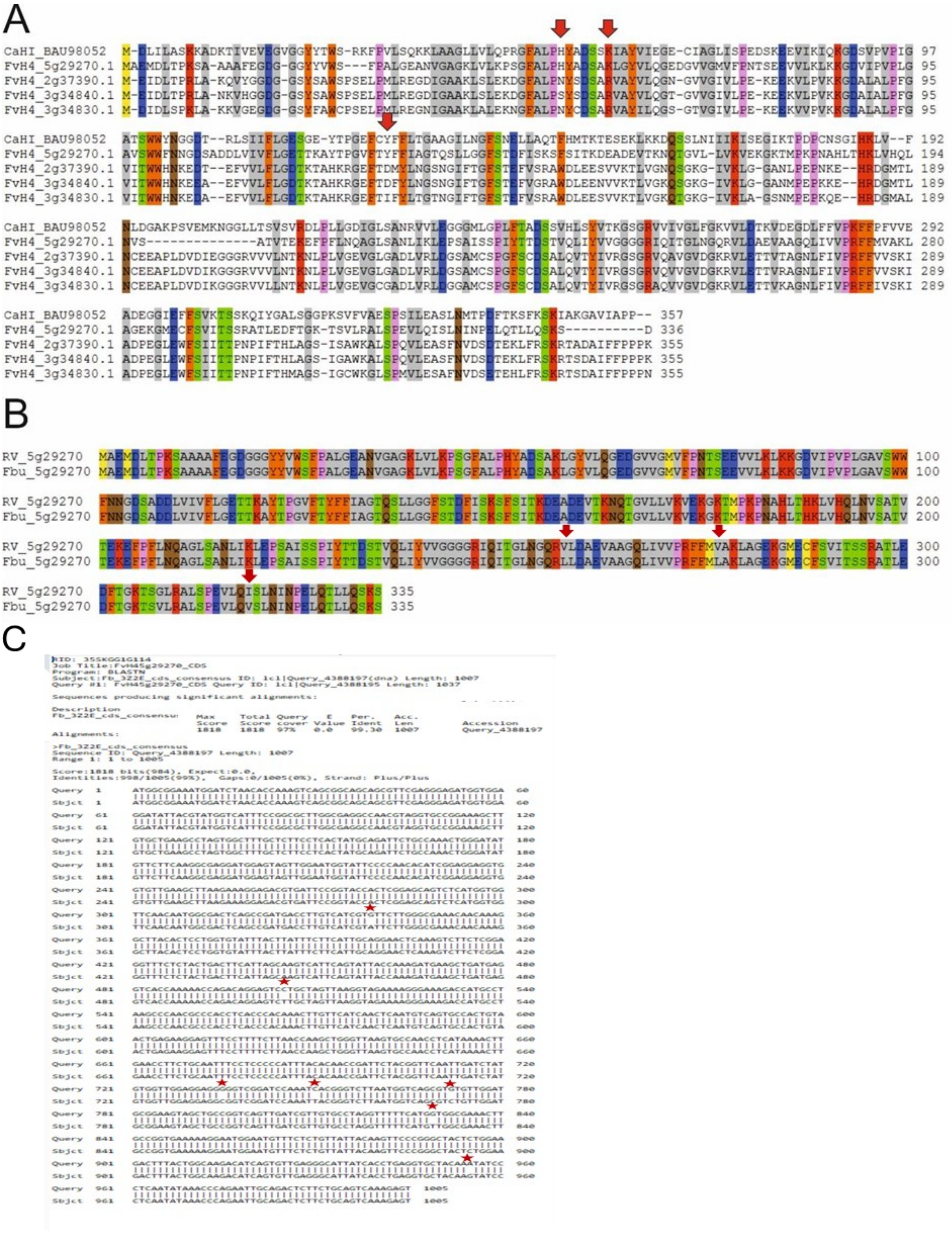

Figure S2. Amino acid alignments of HI and HI-like proteins from *F. vesca* and *F. bucharica*. A) Alignment of four *F. vesca* HI proteins with bell pepper HI (CaHI_BAU98052). The three functionally essential amino acids (H - K- Y) are highlighted by red arrows. Only the *Fragaria* protein 5g29270 possesses all three essential amino acids. B) Alignment of translated coding sequences from the recurrent parent RV and the donor parent *F. bucharica*. Amino acid differences are highlighted by red arrows. C) Alignment of coding sequences (differences highlighted by stars).

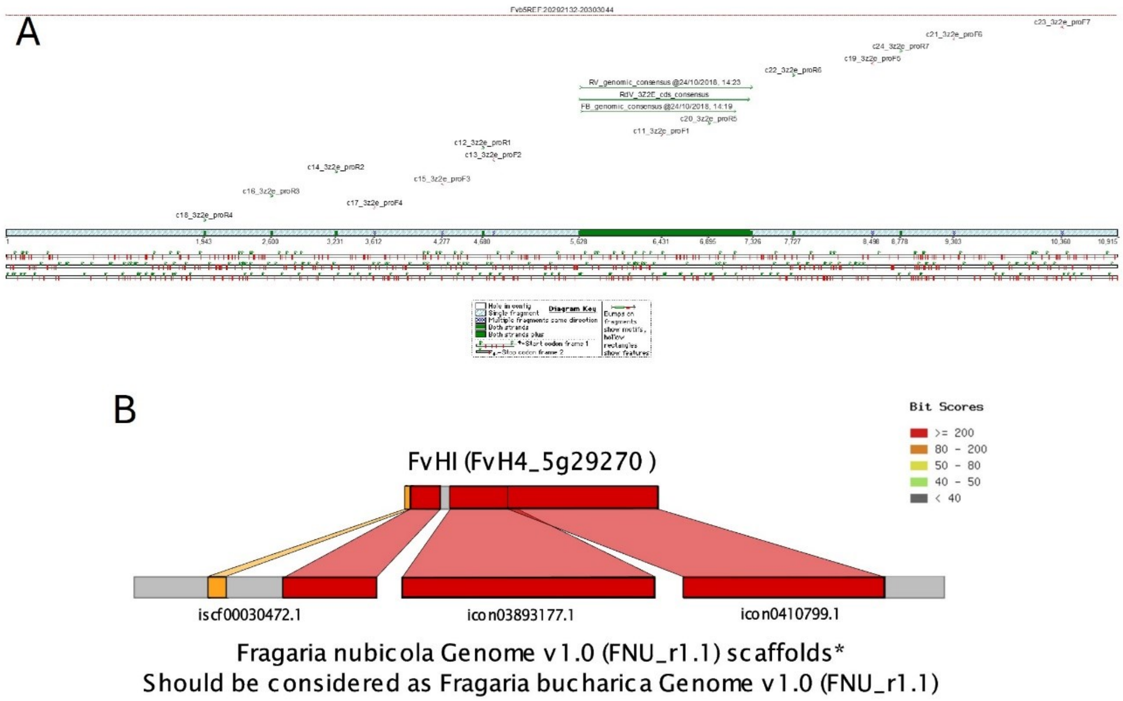

Figure S3. A) Schema promotor amplification. B) Alignment between FvHI regions on *F. vesca* and *F. bucharica* genomes. Reconstruction of BLAST result using the genetic sequence of the *F. vesca* hexenal isomerase gene (FvH4_5g29270) against the *F. nubicola* Genome v1.0 (FNU_r1.1) scaffolds* from the Genome Database for Rosaceae (GDR), in order to improve visualization.

*The genome was reported as *F. nubicola* in Hirakawa et al 2014 based on a USDA accession CFRA 522= PI551853. This accession was originally distributed as *F. nubicola* but subsequently re-identified as *F. bucharica* (Tennessen et al., 2014, doi:10.1093/gbe/evu261; https://www.rosaceae.org/Analysis/500)

Table S1. Near-isogenic lines used in the experiments.

NIL name	1st marker (reference)	1st marker position on LG5 (Mb*)	2nd marker (reference)	2nd marker position on LG5 (Mb*)
LG5:0-4**	CFV-3072	0.06	EMFvi108	0.05
LG5:0-11	CFV-3072	0.06	CFV-3132	1.4
LG5:0-20	CFV-3072	0.06	CEL2	2.6
LG5:0-35	CFV-3072	0.06	FvH4095	5.7
LG5:11-35	CFV-3132	1.4	FvH4095	5.7
LG5:11-76	CFV-3132	1.4	EMFv024	23.0
LG5:20-76	CEL2	2.6	EMFv024	23.0
LG5:39-76	?		EMFv024	23.0
LG5:41-76	UDF009	8.6	EMFv024	23.0
LG5:50-76	?		EMFv024	23.0
LG5:0-76	CFV-3072	0.06	EMFv024	23.0

*megabase pairs of the *F. vesca* genome V4, pseudochromosome 5. Marker locations were determined by using the primer sequences as queries for a BLAST search against the *F. vesca* genome V4

** NIL nomenclature is based on the linkage group positions (in cMs) of the flanking markers.

Table S2. Accession numbers for proteins used for phylogenetic tree construction.

Abbreviation	Plant species	Accession number	HI activity demonstrated	Reference
AtGermin1	*Arabidopsis thaliana*	NP_187070.1		
AtHI-like1	*Arabidopsis thaliana*	NP_180436.1	inactive	Kunishima et al. (2016)
CapaHI	*Capsicum annuum*	XP_016539087.1	active	Kunishima et al. (2016)
CamsHI	*Camellia sinensis*	XP_028056226	active	Chen et al. (2022)
Cs11S	*Cucumis sativus*	XP_011651441.2		
CsHI-1	*Cucumis sativus*	XP_004151504.1	active	Spyropoulou et al. (2017)
CsHI-2	*Cucumis sativus*	Cucsa.078390 *	active	Spyropoulou et al. (2017)
CsHI-like 1	*Cucumis sativus*	Cucsa.033080 *	inactive	Spyropoulou et al. (2017)
CsHI-like 2	*Cucumis sativus*	XP_004150394.1	inactive	Spyropoulou et al. (2017)
FvHI	*Fragaria vesca*	FvH4_5g29270**	active	This work
FvHI-like1	*Fragaria vesca*	FvH4_2g37390**		
FvHI-like2	*Fragaria vesca*	FvH4_3g34840**		
FvHI-like3	*Fragaria vesca*	FvH4_3g34830**		
MtGermin	*Medicago truncatula*	XP_013470283.1		
MtHI-like1	*Medicago truncatula*	XP_003607149.1		
MtHI-like2	*Medicago truncatula*	XP_003605501.1		
MtHI1	*Medicago truncatula*	XP_003629975.1		
MtVicilin	*Medicago truncatula*	XP_003624146.3		
OsGermin	*Oryza sativa*	NP_001395987.1		
OsHI1	*Oryza sativa*	NP_001063876.1	active	Kunishima et al. (2016)
OsHI-like1	*Oryza sativa*	XP_015639453.1		
SlHI-like1	*Solanum lycopersicum*	XP_004229944.1	inactive	Kunishima et al. (2016)
Sl11S1	*Solanum lycopersicum*	XP_004247523.1		
SlLegumin	*Solanum lycopersicum*	XP_004234041.1		
SlVicilin	*Solanum lycopersicum*	NP_001308118		
St11S1	*Solanum tuberosum*	XP_006351693.1		
StGermin	*Solanum tuberosum*	NP_001275369.1		
StHI1	*Solanum tuberosum*	XP_006349431.1	active	Kunishima et al. (2016)
StHI2	*Solanum tuberosum*	XP_006349432.1	active	Kunishima et al. (2016)
StLegumin	*Solanum tuberosum*	XP_006356113.1		
VvGermin	*Vitis vinifera*	NP_001267944.1		

Accession numbers in the NCBI database unless stated otherwise;
* accession number from Phytozome 13 Plant Genomics Database;
** accession number from Genome Database for Rosaceae, *Fragaria vesca* genome V4.a2

Table S3A. Primers used in the experiments.

Primers for	Forward primer	F sequence	Reverse primer	Reverse sequence	Published in
Amplifying *FvHI* for TOPO cloning	5G29270-F	ATGGCGGAAATGGATCTAACACC	5G29270-R	ACAGACTTCGATTGATGGTGCAGG	This work
qRT-PCR	MSI1-F	TCTCCACACCTTTGATTGCCA	MSI1-R	ACACCATCAGTCTCCTGCCAAG	Mouhu et al (2009)
qRT-PCR	Z3E2-F	GAGGGAGATGGTGGAGGATA	Z3E2-R	ACCACCTCCTCCGATGTGT	This work
Gateway primers for overexpression	attB1_5G29270-F	AAAAAGCAGGCTTCGAAGGAGAT AGAACCATGGCGGAAATGGATCTA ACACC	attB2_5G29270-R	AGAAAGCTGGGTACAGACTTCGATTGATG GTGCAGG	This work

Table S3B. Primers used to promotor amplification, designed in this work.

Forward primer	F sequence	Reverse primer	Reverse sequence
c11_3z2e_proF1	TTGAACCACCATGAGACTGC	c12_3z2e_proR1	CATCATCAGGCAACAACAGG
c13_3z2e_proF2	TTCTAGACGCCAACGATGC	c14_3z2e_proR2	TGGGGTATCGTTTCCTACTCC
c15_3z2e_proF3	GAAAATCGGCCTCTTACTCG	c16_3z2e_proR3	ATACACCTGCCCTCTGTTCC
		c18_3z2e_proR4	GGAACCACTCGTTCACAAGC
c19_3z2e_proF5	GACTGCGAGAGTTCGAGAGC	c20_3z2e_proR5	CACGGGTCTTAATGGTCAGC
c21_3z2e_proF6	CTGAAAGCTCGACACACTGC	c22_3z2e_proR6	AAGTATTGGCCAGGATTTGG
c23_3z2e_proF7	GTCCGATGACAACTCAATGC	c24_3z2e_proR7	AAGGCTCTTCCTGGAATTGG
c27_3z2e_proF8	ACTCGGCGATGAGTGTGAG	c25_3z2e_proR8	GGTTGAACGGGTGAGACC
		c26_3z2e_proR9	CTCGTATACACCTGCCCTCTG
		c28_3z2e_proR10	CTTCTTGGAATTGGGGTTTC

Table S4. *F. vesca* proteins with the highest similarity to cucumber (Z)-3:(E)-2-hexenal isomerase.

BLAST hit	e-value	Location in *F. vesca* genome V4
FvH4_5g29270	2.123 e-97	chr5: 20294931..20297997
FvH4_2g37390	2.098 e-67	chr2: 27271351..27272846
FvH4_3g34840	3.436 3-67	chr3: 30118297..30121482
FvH4_3g34830	2.290 e-63	chr3: 30110113..30114706
