# Supplementary figures and images for: The role of Fragaria vesca homolog of a (Z)-3:(E)-2-hexenal isomerase in the development of green-leafy fruit aroma
Source: Hortic Res. 2025 Jun 26;12(10):uhaf163. doi: 10.1093/hr/uhaf163 (PMC12528648; doi:10.1093/hr/uhaf163)

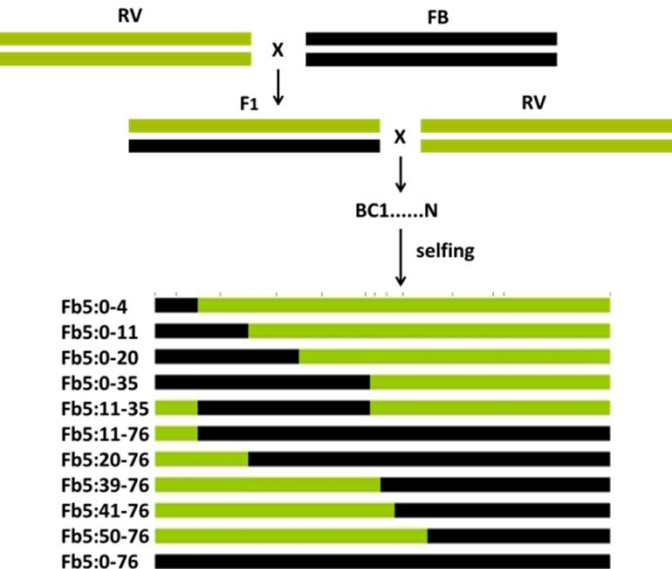

Supplement: Web_Material_uhaf163 [file web_material_uhaf163.zip › Figure_S1.pdf]
